# Significances of Collimator Angle Rotation and Different Angle Combinations in Volumetric-Modulated Arc-Based Stereotactic Radiosurgery With 5-mm Leaf-Width Multileaf Collimator for Single Brain Metastases

**DOI:** 10.7759/cureus.77946

**Published:** 2025-01-24

**Authors:** Kazuhiro Ohtakara, Kojiro Suzuki

**Affiliations:** 1 Department of Radiation Oncology, Kainan Hospital Aichi Prefectural Welfare Federation of Agricultural Cooperatives, Yatomi, JPN; 2 Department of Radiology, Aichi Medical University, Nagakute, JPN

**Keywords:** brain metastases, collimator angle, dose conformity, dose gradient, dose inhomogeneity, multileaf collimator, non-coplanar arc, single brain metastasis, stereotactic radiosurgery, volumetric-modulated arc therapy

## Abstract

Purpose

In linac-based stereotactic radiosurgery (SRS) using a multileaf collimator (MLC) for multiple brain metastases (BMs), the appropriate MLC angle rotation is useful to minimize the intervening brain dose by avoiding island blocking between the lesions localized along the direction of leaf movement. However, in some linac systems, the MLC angle cannot be rotated, or the cephalad rotation range of non-coplanar arcs (NCAs) is severely limited. In addition, the significance of MLC angle rotation has yet to be clarified in SRS for single BMs. This planning study was therefore conducted to investigate the significances of MLC angle rotations and the different angle combinations in SRS with a 5-mm leaf-width MLC for single BMs using volumetric-modulated arcs (VMA) consisting of one coplanar arc and two NCAs rotated 60º cranially.

Materials and methods

The study subjects were 30 lesions in 27 patients with the gross tumor volume (GTV) ranging from 0.08 cc to 48.09 cc (median 9.81 cc). Four VMA-based SRS plans were created for each GTV using an Agility^®^ MLC (Elekta AB, Stockholm, Sweden) and a planning system Monaco^®^ (Elekta AB). The VMA optimization was based on a previously established method with identical settings except for the MLC angle rotation for the three arcs: no rotations (all 0º, All_0), all 90º (All_90), all 45º (All_45), and a combination of 0º, 45º, and 90º (0_45_90). 43.000 Gy in five fractions was uniformly prescribed to each GTV *D*_V-0.01 cc_, the minimum dose to encompass the GTV minus 0.01 cc (*D*_>95%_), for GTV >0.20 cc or *D*_95%_ for GTV ≤0.20 cc. The planning parameters of a total of 120 plans were compared.

Results

There was no significant difference in any parameter between the four groups in Friedman's test. The individual comparisons between the two groups using the Wilcoxon signed-rank test revealed significant differences only in the following four parameters: (1) the GTV coverage by the *D*_eIIV_, the minimum dose of the irradiated isodose volume (IIV) equivalent to each target volume; (2) the *D*_eIIV_ at 2 mm outside the GTV boundary; (3) the IIV spillage receiving ≥75% of the prescribed dose; and (4) the *D*_eIIV_ coverage value at 2 mm inside the GTV boundary. Overall, the All_45 showed the best tendency between the three groups with the specific fixed MLC angle. Regarding the prescribed isodose volume (PIV) spillage outside the GTV, the median and third quartile values were smallest in the All_45, and the maximum value ​​was smallest in the 0_45_90. The maximum difference in the PIV spillage was 0.52 cc between the 0_45_90 (smallest) and the All_45.

Conclusions

An MLC angle rotation and the different combinations can improve dose distribution in VMA-based SRS even for single BMs, and the optimal rotation angle differs for individual lesions. VMA without MLC angle rotation (all 0º) can provide an overall non-inferior dose distribution in the three-arc arrangement that divides the cranial hemisphere into thirds. When selecting a fixed MLC angle for efficient irradiation, 45º is recommended among 0º, 90º, and 45º. Further study is required to find criteria for determining the appropriate MLC angle for each lesion.

## Introduction

Stereotactic radiosurgery (SRS) plays an increasing role in the management of patients with brain metastases (BMs) as the prognosis improves due to advances in systemic therapy [1,2]. Image-guided frameless SRS facilitates daily fractionated irradiation and has contributed to improved safety and expanded indications for large lesions [3]. It is important to appropriately perform SRS using a general-purpose linac equipped with a standard 5-mm leaf-width multileaf collimator (MLC) to enable more patients with BMs to benefit from SRS [4]. Inverse planning-based dynamic conformal arcs (DCA) and volumetric-modulated arcs (VMA) are useful in providing effective and efficient linac-based SRS, especially for multiple lesions [4,5]. In simultaneous irradiation for multiple lesions, the proper MLC angle rotation, along with the arc arrangement including multiple non-coplanar arcs (NCAs), is useful to sufficiently reduce the intervening brain dose by avoiding island blocking between the lesions localized along the direction of leaf movement [6-12]. In addition, an arc arrangement consisting of one coplanar arc and two NCAs, arranged to divide the cranial hemisphere into thirds, is useful for achieving steep dose gradients outside and inside the gross tumor volume (GTV) boundary while maintaining irradiation efficiency [13]. In the three-arc arrangement, each arc can have a different MLC angle. Even with a coplanar arc-only arrangement, dose distribution equivalent to that achieved with a <5-mm leaf-width mini-MLC can be achieved by setting the MLC angles to be 90º different as a double reciprocating arc [13,14]. Thus, a standard linac has a high degree of freedom in setting the MLC angle and the rotation range of the NCAs for intracranial SRS. However, in some linac systems specialized for high-precision irradiation, the MLC angle cannot be rotated, or the cephalad rotation range of NCAs is severely limited [15-18].

Although there is no island blocking problem when irradiating a single BM, the significances of MLC angle rotations or the different combinations for single BMs have yet to be clarified, especially for the arc arrangement including at least two NCAs. The combination of 5-mm leaf-width MLC angles that differ by 90º may improve the dose distribution even for a single lesion by creating an irradiation field that is virtually equivalent to that with a 2.5-mm leaf-width MLC.

This planning study was therefore conducted to investigate the significances of MLC angle rotations and the different combinations in VMA-based SRS using a 5-mm leaf-width MLC for single BMs. Specifically, for all the MLC angle settings of 0º, the significances of rotating it by 90º or 45º and the combination of 0º, 45º, and 90º were examined in the NCAs involving three-arc arrangement.

## Materials and methods

This study was approved by the Clinical Research Review Board of Kainan Hospital Aichi Prefectural Welfare Federation of Agricultural Cooperatives (approval number: 20240830-01).

The study subjects were 30 lesions in 27 patients, all of which underwent multi-fraction SRS at our facility, and overlapped with the previous study subjects [19]. Each lesion was treated as a single brain metastasis. Each GTV was contoured after image co-registration and fusion using the MIM Maestro^®^ Version 7.1.3 (MIM Software Inc., Cleveland, Ohio, United States) as described previously [20,21]. The GTV ranged from 0.08 cc to 48.09 cc (median value: 9.81 cc; interquartile range (IQR): 4.38, 24.31 cc).

The linac used was the Infinity^®^ (Elekta AB, Stockholm, Sweden) equipped with a 5-mm leaf-width MLC Agility® (Elekta AB), which provides a flattening filter-free (FFF) mode of a 6-MV X-ray beam. The Monaco^®^ Version 5.51.10 (Elekta AB) was used for VMA planning [4,22]. The irradiation isocenter was uniformly set at each GTV center. Three arcs were uniformly allocated for each GTV, which consisted of one coplanar arc with 360º rotation and two NCAs with each 180º rotation to divide the cranial hemisphere into thirds [13]. In this study, the MLC angle of each arc was set in four patterns with each abbreviation in parentheses: all 0º (All_0), all 90º (All_90), all 45º (All_45), and a combination of 0º, 45º, and 90º (0_45_90). In the 0_45_90, the MLC angles of 45º and 90º were applied to the left and right NCAs, respectively. The increment parameter was uniformly set to 20º per each arc [23].

Each VMA planning was uniformly optimized in the Pareto mode to maximize the steepness of dose gradient outside the GTV boundary [22]. Three physical cost functions with identical settings were applied to each GTV and the patient's head contour as described previously [22]. A commonly prescribed dose of 43.000 Gy in five fractions was assigned to each GTV *D*_V-0.01 cc _(*D*_>95%_), the minimum dose to encompass each GTV minus 0.01 cc, for GTV >0.20 cc or *D*_95%_ for GTV ≤0.20 cc, to minimize the uncovered GTV to the equivalent of a ≤3-mm-diameter lesion, based on a previous study [22,24]. Consequently, the GTV coverage values by the *D*_V-0.01 cc_ ranged from 95.00% to 99.98% (median value: 99.90%; IQR: 99.77, 99.96%) [19]. However, in actual practice, this dose is applied to a GTV of ≤8.5 cc to ensure safety [24]. In setting the intensity-modulated radiation therapy (IMRT) prescription parameters, the beamlet width, the target margin, and the avoidance margin were set as 0.30 cm, normal (8 mm), and normal (8 mm), respectively. In setting the sequencing parameters, the segment shape optimization was enabled with the high-quality leaf positions of 20, which prioritizes the quality of planning over the time required for planning. The maximum control points per arc, the minimum segment width, and the fluence smoothing were uniformly set as 1024, 0.5 cm, and the medium, respectively [22]. In setting the calculation properties, the grid spacing and the statistical uncertainty of an X-ray voxel Monte Carlo algorithm were set as 1 mm and 1.00% per calculation, respectively, choosing the dose deposition to the medium [19]. After the completion of the optimization, each GTV coverage with 43.000 Gy was rescaled according to each coverage value of ≥95% [24]. A change in the GTV dose after the rescaling was recorded as the rescaling ratio [22].

In comparisons of the plan quality, the superiority of dose distribution was evaluated based on the following indicators in order of priority: dose conformity to the GTV, the steepness of dose gradient outside the GTV along with the appropriateness of dose attenuation margin outside the GTV surface, the steepness of dose increase 2-4 mm inside the GTV boundary, and the concentric lamellarity of dose gradients outside and inside the GTV boundary [22]. Isotropic margins of 2 mm, -2 mm, and -4 mm were added to each GTV surface using MIM Maestro^®^ to create the GTV + 2 mm, GTV - 2 mm, and GTV - 4 mm structures, respectively [22,25]. The GTV - 2 mm and GTV - 4 mm were created only for GTVs of ≥0.72 cc (28 lesions) and ≥2.20 cc (26 lesions), respectively, to ensure the minimum meaningful volumes for evaluation [25]. The irradiated isodose volumes (IIVs), including the GTV, receiving ≥100% (prescribed isodose volume (PIV)), ≥75%, and ≥50% of the prescribed dose, i.e., ≥43.000 Gy, ≥32.250 Gy, and ≥21.500 Gy, were calculated from the dose-volume histogram (DVH) as described previously [22,26]. The IIVs may include normal tissues, e.g., the cerebrospinal fluid space, other than the brain parenchyma. The absolute volumes obtained by subtracting the GTV from these IIVs were recorded as each spillage volume: i.e., 100%, 75%, and 50% PIV spillage [22]. The near-maximum dose (*D*_near-max_) of each GTV was recorded as the minimum dose encompassing 0.01 cc of the GTV (*D*_0.01 cc_) for GTV ≥0.20 cc or *D*_5%_ (*D*_<0.01 cc_) for GTV <0.20 cc [22]. The GTV dose inhomogeneity was recorded as the GTV *D*_V-0.01 cc_ (%) relative to the GTV *D*_near-max_ (100%) [22]. The near-minimum doses of GTV + 2 mm, GTV, GTV - 2 mm, and GTV - 4 mm were evaluated as each *D*_eIIV_, the minimum dose to encompass the IIV equivalent to a target volume on the DVH [25,27]. Each *D*_eIIV_ was recorded as the relative dose to the prescribed dose (100%) [25,27].

The GTV dose conformity was evaluated using the smallness of 100% PIV spillage (cc), the closeness of GTV *D*_eIIV_ to the prescribed dose, and the high GTV coverage value by the *D*_eIIV_ [19,22,24]. The steepness of dose gradient outside the GTV was evaluated using the smallness of 75% and 50% PIV spillage (cc) [22]. The appropriateness of dose attenuation margin was compared using the GTV + 2 mm *D*_eIIV_ relative to the prescribed dose: basically, the lower the dose, the more appropriate it is; however, it must not be too low for small lesions [27]. The steepness of dose increase inside the GTV boundary was evaluated using the *D*_eIIV_s (%) of the GTV, GTV - 2 mm, and GTV - 4 mm [25]. The concentric lamellarity of dose gradients outside and inside the GTV boundary was evaluated using the coverage values of GTV + 2 mm, GTV -2 mm, and GTV - 4 mm by each *D*_eIIV_ [25,27].

For statistical analyses, paired nonparametric tests were adopted, considering the dominant distributions of variables based on the normality test results by the Shapiro-Wilk test. Box-and-whisker plots (BWPs) were used to show the distribution of each variable. In the BWP, the whiskers indicate the nearest values ≤1.5 times the IQR. The cross marks beyond the lines denote the outliers >1.5 times the IQR. Friedman's test (FT) and Scheffe's post hoc test (SPHT) were applied to compare three numerical variables. The Wilcoxon signed-rank test (WSRT) was used to compare two numerical variables. If there was no significant difference between the two numerical variables in the SPHT with the p-value being <0.9, the WSRT was additionally applied to compare them further. Statistical significance was considered at p<0.05 and expressed on a three-level scale: p<0.05 (*), p<0.01 (**), and p<0.001 (***). All analyses were performed using a dedicated software BellCurve for Excel^®^ (Version 4.05; Social Survey Research Information Co., Ltd., Tokyo, Japan).

## Results

The normality of the GTV distribution was significantly dismissed by the Shapiro-Wilk test (p=0.010).

The results of the planning and dosimetric comparisons between the four groups are shown in Tables 1-2, along with the results of FT, SPHT, and WSRT.

**Table 1:** Dosimetric comparisons between the four different collimator angle settings: part 1. Representative values, e.g., IQR, ​​other than the median are shown separately as a box-and-whisker plot in each figure. If the p-value of SPHT is <0.9, the result of the WSRT is added. Statistical significance is displayed in three levels: *p<0.05, **p<0.01, and ***p<0.001. PIV: prescribed isodose volume; GTV: gross tumor volume; *D*_eIIV_: the minimum dose to encompass the irradiated isodose volume equivalent to a reference target volume; X% PIV: the volume irradiated with ≥X% of the prescribed dose, including a target volume; FT: Friedman's test; SPHT: Scheffe's post hoc test; WSRT: Wilcoxon signed-rank test; NS: not significant; IQR: interquartile range; NR: not required; All_0: all collimator angles of 0º; All_90: all collimator angles of 90º; All_45: all collimator angles of 45º; 0_45_90: collimator angles of 0º, 45º, and 90º

Parameter			Rescaling ratio	PIV spillage (cc)	GTV *D*_eIIV_ coverage (%)	GTV + 2 mm *D*_eIIV_ (%)	GTV + 2 mm *D*_eIIV_ coverage (%)	75% PIV spillage (cc)	50% PIV spillage (cc)
FT	P-value		0.326 NS	0.350 NS	0.132 NS	0.155 NS	0.185 NS	0.116 NS	0.731 NS
	Chi-squared value		3.460	3.281	5.620	5.248	4.833	5.920	1.294
SPHT (WSRT)	All_0 vs All_90	Median values	1.032	1.48	97.02	81.31	97.17	7.24	17.92
			1.031	1.45	96.98	80.86	97.05	7.18	17.76
		P-value	0.881 NS	0.948 NS	0.396 NS	0.750 NS	0.688 NS	0.581 NS	0.905 NS
		Chi-squared value	0.669	0.361	2.969	1.214	1.474	1.960	0.564
		WSRT	0.392 NS	NR	0.007 **	0.033 *	0.289, NS	0.299, NS	NR
	All_0 vs All_45	Median values	1.032	1.48	97.02	81.31	97.17	7.24	17.92
			1.032	1.38	97.02	80.87	97.13	7.05	17.57
		P-value	0.652 NS	0.381 NS	1.0000 NS	0.166 NS	0.251 NS	0.262 NS	0.905 NS
		Chi-squared value	1.633	3.073	0.003	5.079	4.096	4.000	0.564
		WSRT	0.596 NS	0.171 NS	NR	0.020 *	0.079 NS	0.030 *	NR
	All_0 vs 0_45_90	Median values	1.032	1.48	97.02	81.31	97.17	7.24	17.92
			1.029	1.43	97.07	80.73	97.14	7.02	17.50
		P-value	0.362 NS	0.776 NS	0.976 NS	0.550 NS	0.983 NS	0.184 NS	0.750 NS
		Chi-squared value	3.201	1.106	0.208	2.110	0.164	4.840	1.214
		WSRT	0.0500 NS	0.258 NS	NR	0.136 NS	NR	0.066 NS	0.177 NS
	All_90 vs All_45	Median values	1.031	1.45	96.98	80.86	97.05	7.18	17.76
			1.032	1.43	97.02	80.87	97.13	7.05	17.57
		P-value	0.976 NS	0.723 NS	0.370 NS	0.723 NS	0.884 NS	0.948 NS	1.0000 NS
		Chi-squared value	0.212	1.327	3.146	1.327	0.655	0.360	0.000
		WSRT	NR	0.254, NS	0.003 **	0.360, NS	0.247, NS	NR	NR
	All_90 vs 0_45_90	Median values	1.031	1.45	96.98	80.86	97.05	7.18	17.76
			1.029	1.43	97.07	80.73	97.14	7.02	17.50
		P-value	0.815 NS	0.977 NS	0.191 NS	0.989 NS	0.884 NS	0.887 NS	0.989 NS
		Chi-squared value	0.943	0.203	4.749	0.123	0.655	0.640	0.123
		WSRT	0.156 NS	NR	0.178 NS	NR	0.770 NS	0.614 NS	NR
	All_45 vs 0_45_90	Median values	1.032	1.43	97.02	80.87	97.13	7.05	17.57
			1.029	1.43	97.07	80.73	97.14	7.02	17.50
		P-value	0.967 NS	0.921 NS	0.983 NS	0.887 NS	0.454 NS	0.998 NS	0.989 NS
		Chi-squared value	0.261	0.492	0.164	0.642	2.621	0.040	0.123
		WSRT	NR	NR	NR	0.689 NS	0.230 NS	NR	NR

**Table 2:** Dosimetric comparisons between the four different collimator angle settings: part 2. Representative values, e.g., IQR, ​​other than the median are shown separately as a box-and-whisker plot in each figure. If the p-value of SPHT is <0.9, the result of WSRT is added. Statistical significance is displayed in three levels: *p<0.05, **p<0.01, and ***p<0.001. GTV: gross tumor volume; *D*_V-0.01 cc_: the minimum dose to cover a target volume minus 0.01 cc; IDS: isodose surface; *D*_eIIV_: the minimum dose to encompass the irradiated isodose volume equivalent to a reference target volume; NS: not significant; NR: not required; FT: Friedman's test; SPHT: Scheffe's post hoc test; WSRT: Wilcoxon signed-rank test; All_0: all collimator angles of 0º; All_90: all collimator angles of 90º; All_45: all collimator angles of 45º; 0_45_90: collimator angles of 0º, 45º, and 90º

Parameter			GTV *D*_V-0.01 cc_ % IDS	GTV *D*_eIIV_ (%)	GTV – 2 mm *D*_eIIV_ (%)	GTV – 2 mm *D*_eIIV_ coverage (%)	GTV – 4 mm *D*_eIIV_ (%)	GTV – 4 mm *D*_eIIV_ coverage (%)
FT	P-value		0.633 NS	0.693 NS	0.702 NS	0.109 NS	0.605 NS	0.742 NS
	Chi-squared value		1.716	1.455	1.414	6.054	1.846	1.246
SPHT (WSRT)	All_0 vs All_90	Median values	58.68	107.10	133.44	95.88	151.59	91.30
			58.78	107.07	134.11	95.37	151.03	89.88
		P-value	1.0000 NS	0.977 NS	0.833 NS	1.0000 NS	0.980 NS	0.991 NS
		Chi-squared value	0.000	0.203	0.868	0.003	0.185	0.104
		WSRT	NR	NR	0.452 NS	NR	NR	NR
	All_0 vs All_45	Median values	58.68	107.10	133.44	95.88	151.59	91.30
			57.99	106.97	135.17	95.91	152.22	91.04
		P-value	0.959 NS	0.989 NS	0.913 NS	0.293 NS	0.864 NS	0.962 NS
		Chi-squared value	0.304	0.123	0.525	3.720	0.739	0.289
		WSRT	NR	NR	NR	0.024 *	0.238 NS	NR
	All_0 vs 0_45_90	Median values	58.68	107.10	133.44	95.88	151.59	91.30
			57.74	106.92	134.87	96.03	152.36	91.58
		P-value	0.905 NS	0.921 NS	1.0000 NS	0.485 NS	0.980 NS	0.937 NS
		Chi-squared value	0.564	0.492	0.000	2.446	0.185	0.415
		WSRT	NR	NR	NR	0.076 NS	NR	NR
	All_90 vs All_45	Median values	58.78	107.07	134.11	95.37	151.03	89.88
			57.99	106.97	135.17	95.91	152.22	91.04
		P-value	0.959 NS	0.887 NS	0.998 NS	0.318 NS	0.980 NS	0.864 NS
		Chi-squared value	0.304	0.642	0.043	3.522	0.185	0.739
		WSRT	NR	0.600, NS	NR	0.014 *	NR	0.258 NS
	All_90 vs 0_45_90	Median values	58.78	107.07	134.11	95.37	151.03	89.88
			57.74	106.92	134.87	96.03	152.36	91.58
		P-value	0.905 NS	0.723 NS	0.833 NS	0.515 NS	0.864 NS	0.817 NS
		Chi-squared value	0.564	1.327	0.868	2.285	0.739	0.935
		WSRT	NR	0.178 NS	0.616 NS	0.032 *	0.732 NS	0.280 NS
	All_45 vs 0_45_90	Median values	57.99	106.97	135.17	95.91	152.22	91.04
			57.74	106.92	134.87	96.03	152.36	91.58
		P-value	0.638 NS	0.989 NS	0.913 NS	0.988 NS	0.646 NS	0.9997 NS
		Chi-squared value	1.696	0.123	0.525	0.133	1.662	0.012
		WSRT	0.147 NS	NR	NR	NR	0.159 NS	NR

**Figure 1:**
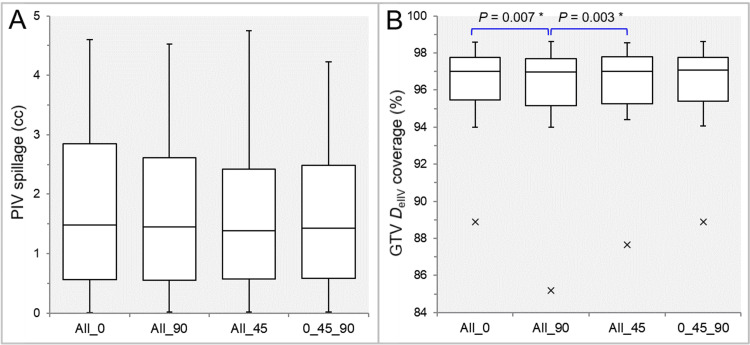
Comparisons of the GTV dose conformity. The images show BWPs (A,B) for comparisons of PIV spillage outside the GTV (A) and the GTV coverage value by the *D*_eIIV_ (B), along with the significant results of WSRT in B, between the four groups. PIV: prescribed isodose volume; *D*_eIIV_: the minimum dose to encompass the irradiated isodose volume equivalent to a reference target volume; All_0: all collimator angles of 0º; All_90: all collimator angles of 90º; All_45: all collimator angles of 45º; 0_45_90: collimator angles of 0º, 45º, and 90º; BWPs: box-and-whisker plots; WSRT: Wilcoxon signed-rank test

There were no significant differences in the rescaling ratios (Table 1). There were no significant differences in the PIV spillage (Table 1, Figure 1A), while the GTV coverage value by the *D*_eIIV_ was significantly smaller in the All_90 than in the All_0 and All_45 (Table 1, Figure 1B).

The maximum differences in the PIV spillage ranged from 0.01 cc to 0.52 cc (median: 0.13 cc; IQR: 0.06, 0.23 cc), in which the maximum volume difference corresponded to a 1-cm-diameter lesion. The maximum difference of 0.52 cc was between the 0_45_90 and All_45 for an irregularly shaped GTV of 35.74 cc. In the PIV spillage, the median and third quartile values were smallest in the All_45, and the maximum value ​​was smallest in the 0_45_90 (Figure 1A).

**Figure 2:**
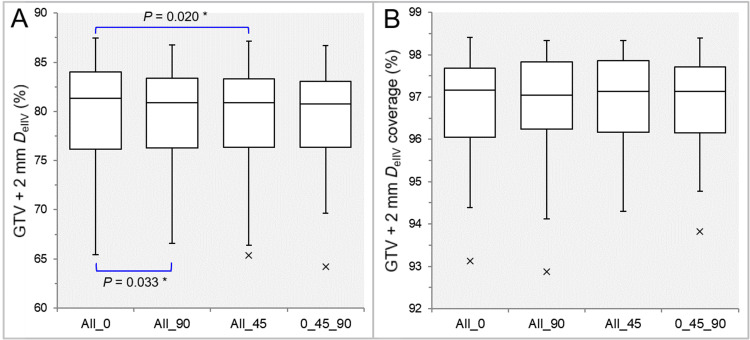
Comparisons of the appropriateness of dose attenuation margin outside the GTV. The images show BWPs (A,B), along with the significant results of WSRT, for comparisons of the GTV + 2 mm *D*_eIIV_ (%) relative to the prescribed dose (100%) (A) and the coverage value of GTV + 2 mm by the *D*_eIIV_ (B) between the four groups. GTV: gross tumor volume; *D*_eIIV_: the minimum dose to encompass the irradiated isodose volume equivalent to a reference target volume; All_0: all collimator angles of 0º; All_90: all collimator angles of 90º; All_45: all collimator angles of 45º; 0_45_90: collimator angles of 0º, 45º, and 90º; BWPs: box-and-whisker plots; WSRT: Wilcoxon signed-rank test

The GTV + 2 mm *D*_eIIV_s were significantly higher in the All_0 than in the All_90 and All_45 (Table 1, Figure 2A), while there was no significant difference in the coverage values of GTV + 2 mm by the *D*_eIIV_ (Table 1, Figure 2B).

**Figure 3:**
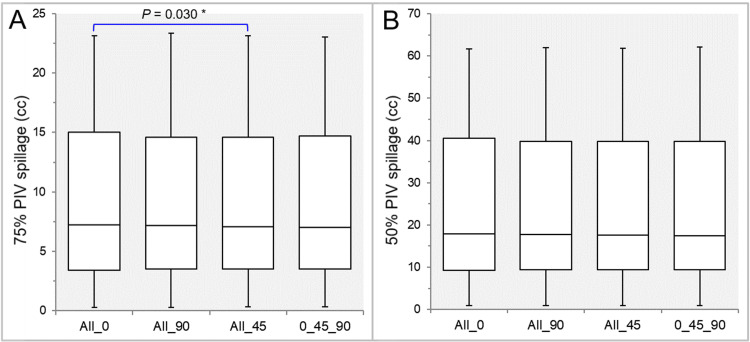
Comparisons of the steepness of dose gradient outside the GTV. The images show BWPs (A,B), along with the significant result of WSRT, for comparisons of 75% (A) and 50% (B) PIV spillage volumes outside the GTV between the four groups. GTV: gross tumor volume; PIV: prescribed isodose volume; X% PIV: the isodose volume irradiated with ≥X% of the prescribed dose, including a target volume; All_0: all collimator angles of 0º; All_90: all collimator angles of 90º; All_45: all collimator angles of 45º; 0_45_90: collimator angles of 0º, 45º, and 90º; BWPs: box-and-whisker plots; WSRT: Wilcoxon signed-rank test

The 75% PIV spillage volumes were significantly smaller in the All_45 than in the All_0 (Table 1, Figure 3A), while the 0_45_90 showed a smaller trend than the All_0 (p=0.066).

In the 75% PIV spillage, the median and maximum values were smallest in the 0_45_90, while the third quartile value ​​was smallest in the All_45 (Figure 3A). There was no significant difference in the 50% PIV (Table 1, Figure 3B). The maximum differences in the 50% PIV spillage ranged from 0.07 cc to 1.99 cc (median: 0.39 cc; IQR: 0.20, 0.55 cc). In the 50% PIV spillage, the median and third quartile values were smallest in the 0_45_90, while the maximum value ​​was smallest in the All_0 (Figure 3B).

**Figure 4:**
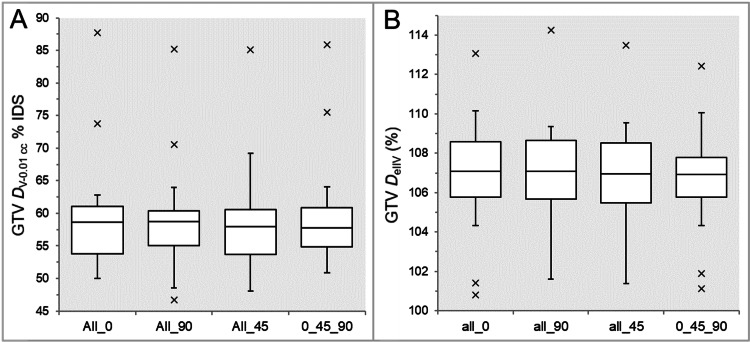
Comparisons of the GTV dose inhomogeneity and the steepness of dose increase just inside the prescribed isodose surface. The images show BWPs (A,B) for comparisons of the prescribed isodose surface (%) relative to the GTV near-maximum dose (100%) (A) and the GTV *D*_eIIV_ (%) relative to the prescribed dose (100%) (B) between the four groups. GTV: gross tumor volume; *D*_V-0.01 cc_: the minimum dose to encompass a target volume minus 0.01 cc; IDS: isodose surface; *D*_eIIV_: the minimum dose to encompass the irradiated isodose volume equivalent to a reference target volume; All_0: all collimator angles of 0º; All_90: all collimator angles of 90º; All_45: all collimator angles of 45º; 0_45_90: collimator angles of 0º, 45º, and 90º; BWPs: box-and-whisker plots

There were no significant differences in the GTV dose inhomogeneities (Table 2 and Figure 4A) and the GTV *D*_eIIV_s (Table 2 and Figure 4B).

**Figure 5:**
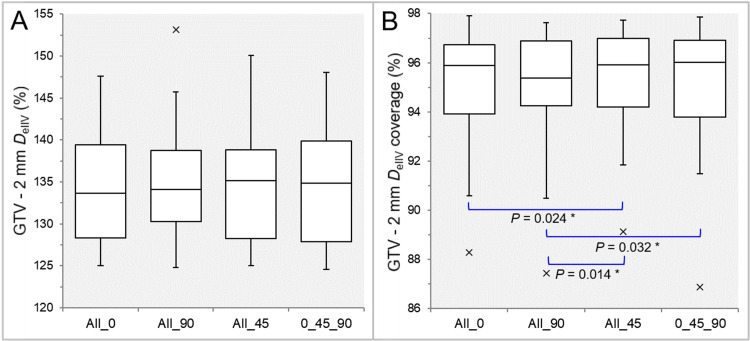
Comparisons of the steepness of dose increase and the concentric lamellarity at 2 mm inside the GTV boundary. The images show BWPs (A,B), along with the significant results of WSRT in B, for comparisons of the GTV - 2 mm *D*_eIIV_ (%) relative to the prescribed dose (100%) (A) and the coverage value of GTV - 2 mm by the *D*_eIIV_ (B) between the four groups. GTV: gross tumor volume; *D*_eIIV_: the minimum dose to encompass the irradiated isodose volume equivalent to a reference target volume; All_0: all collimator angles of 0º; All_90: all collimator angles of 90º; All_45: all collimator angles of 45º; 0_45_90: collimator angles of 0º, 45º, and 90º; BWPs: box-and-whisker plots; WSRT: Wilcoxon signed-rank test

There was no significant difference in the GTV - 2 mm *D*_eIIV_ (Table 2 and Figure 5A).

The coverage values of GTV - 2 mm by the *D*_eIIV_ were significantly lower in the All_90 than in the All_45 and the 0_45_90, while those were also significantly smaller in the All_0 than in the All_45 (Table 2 and Figure 5B). There was no significant difference between the All_45 and 0_45_90 (Table 2 and Figure 5B).

**Figure 6:**
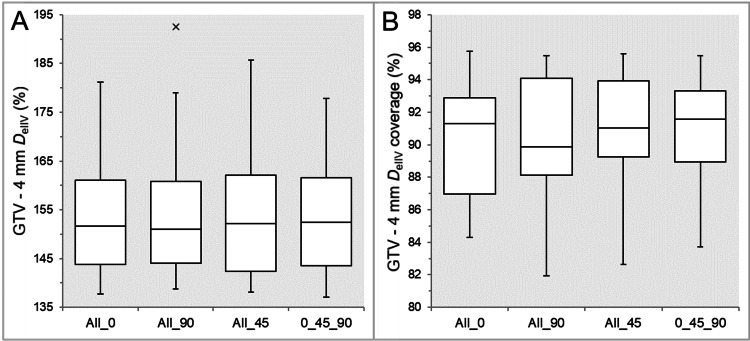
Comparisons of the steepness of dose increase and the concentric lamellarity at 4 mm inside the GTV boundary. The images show BWPs (A,B) for comparisons of the GTV - 4 mm *D*_eIIV_ (%) relative to the prescribed dose (100%) (A) and the coverage value of GTV - 4 mm by the *D*_eIIV_ (B) between the four groups. GTV: gross tumor volume; *D*_eIIV_: the minimum dose to encompass the irradiated isodose volume equivalent to a reference target volume; All_0: all collimator angles of 0º; All_90: all collimator angles of 90º; All_45: all collimator angles of 45º; 0_45_90: collimator angles of 0º, 45º, and 90º; BWPs: box-and-whisker plots

There were no significant differences in the GTV - 4 mm *D*_eIIV_ (Table 2 and Figure 6A) and the coverage values of GTV - 2 mm by the *D*_eIIV_ (Table 2 and Figure 6B).

Taken together, there was no significant difference in any parameter between the four groups in FT. The individual comparisons between the two groups using WSRT revealed significant differences only in the following four parameters: (1) the GTV coverage by the *D*_eIIV_; (2) the GTV + 2 mm *D*_eIIV_; (3) the 75% PIV spillage; and (4) the coverage value of GTV - 2 mm by the *D*_eIIV_. Overall, the All_45 showed the best tendency between the three groups fixed at the specific MLC angle.

## Discussion

This study was conducted with the expectation that the All_90 or All_45 would be better than the All_0 and that the 0_45_90 would create the best dose distribution [13]. However, the results were unexpected, and no specific MLC angle setting was outstandingly superior. In the comparisons across the 30 lesions, there were significant statistical differences in some parameters; however, the differences were not considered to be large enough to make a major difference in the clinical outcomes, compared to the differences in the optimization method and prescription dose. VMA-based SRS without the MLC angle rotation (all 0º) generally provides non-inferior dose distribution and may be an option if priority is given to starting irradiation early from image acquisition or shortening the delivery time. This non-inferiority was likely attributed to the arc arrangement that fully and effectively utilizes the cephalad hemisphere space: the arrangement to divide the hemisphere into thirds with sufficient rotation of each arc with 180-360º [13]. In cases with only coplanar arcs or NCAs that are severely restricted in cephalad rotation, it may be advantageous to combine multiple different MLC angles [9,11]. In addition, the optimization method specific to Monaco® that effectively utilizes the dynamic jaw movement beyond the tracking to form the irradiation field also likely contributed to reducing the effects of MLC angle rotations on dose distribution.

For individual lesions, differences in the MLC angles led to definite differences in the dose distribution, and the optimal angle varied from lesion to lesion. Among the various parameters for evaluating dose distribution, the most important is dose conformity to the GTV, and as an evaluation index, PIV spillage is more important than common conformity indices that appear to have better values as the tumor increases [23,26,28]. Especially with the dose prescription to GTV *D*_V-0.01 cc_, the GTV over-coverage with the PIV tends to be excessive, so minimizing the PIV spillage is the top priority [22,24]. From the median to the third quartile of the PIV spillage, the All_45 and 0_45_90 were smaller than the All_0 and All_90, while the maximum value was the highest for the All_45 and the lowest for the 0_45_90. Based on the results, we recommended 45º when fixing the MLC angle and decided to continue using the 0_45_90 as the template we usually use.

The great strength of a standard linac is the high degree of freedom in the MLC angle rotation and its combination as well as the cranial rotation range of NCAs. Ensuring and verifying the irradiation accuracy when using NCAs is a major issue in a general-purpose linac; however, it can be solved by leveraging an image guidance system for bone structure and/or body surface [29,30]. Setting the MLC angle is an important element in efficiently creating the optimal dose distribution for each lesion even in single BMs. In the future, it is expected that advancements in planning systems will make it possible to automatically select the optimal MLC angle based on the shape of each lesion. In particular, a format that allows to specify either all the same angles or a combination of different angles is desirable.

Study limitations

In this study, the MLC angle selection is limited to 0º, 45º, and 90º and did not include other angles such as 15º or 30º. The optimal MLC angle can be estimated by carefully checking the beam's eye views as the gantry rotates. Additionally, this study did not include comparisons based on actual dosimetry. The current study with 30 lesions is insufficient, and the optimal MLC angle should be determined using more lesions based on differences in volume, shape characteristics, and localization.

## Conclusions

An MLC angle rotation and the different combinations can substantially improve dose distribution in VMA-based SRS even for single BMs, and the optimal rotation angle differs for individual lesions. With optimization using Agility^®^ and Monaco^®^, VMA without the MLC angle rotation (all 0º) can provide overall non-inferior dose distribution in the three-arc arrangement that divides the cranial hemisphere into thirds. When selecting a fixed MLC angle for efficient irradiation, 45º is recommended among 0º, 90º, and 45º. Further study is required to find criteria for determining the appropriate MLC angle for each lesion.
